# 

**Published:** 1956-07

**Authors:** 


MEDICAL JOURNAL OF THE SOUTH-WEST

*(Incorporating the Bristol Medico-Chirurgical Journal)*

Established 1883

VOL. 71 (iii) July, 1956 NO. 261

### Contents

*PUBLISHED QUARTERLY*

*ANNUAL SUBSCRIPTION* 16/- *POST FREE*

PUBLISHED UNDER THE AUSPICES OF THE
BRISTOL MEDICO-CHIRURGICAL SOCIETY

### *Editorial Committee*

Dr. J. E. Cates, Department of Medicine, Bristol Royal Infirmary. *Editor.*
Dr. A. H. Gale, The University, Bristol 8. *Assistant Editor.*
Dr. J. Macrae, Ham Green Hospital. *Treasurer.*
Dr. N. J. Brown, Southmead Hospital, Bristol.
Mr. J. P. Partridge, Southmead Hospital, Bristol.

### *Local Correspondents*

*Bath* . . Mr. A. D. Bateman, 3 The Circus, Bath.
*Exeter* . . Dr. L. N. Jackson, Mount Jocelyn, Crediton, Devon.
*Gloucester* . Dr. R. F. Jarrett, 9 College Green, Gloucester.
*Plymouth* . Dr. C. R. Croft, Lexden, Hartley Av., Plymouth.
*Taunton* . Dr. M. McAlley, 1 The Crescent, Taunton.
*Torquay* . Dr. A. Robinson Thomas, 20 Devon Square, Newton Abbot, Devon.
*Truro* . . Dr. F. D. M. Hocking, St. Andrews, Perranporth, Cornwall.
*Yeovil* . . Mr. J. A. Vere Nicoll, Knapp House, Yeovil, Somerset.

### NOTICE TO CONTRIBUTORS

Original articles are invited, provided they have not been published elsewhere. Notes of forthcoming events, meetings held, degrees conferred, staff appointments, and other local news are welcomed. The editorial committee reserves the right to make literary corrections.

Illustrations should always be supplied ready for reproduction. All re-touchings or other operations required will be charged to the author. Line drawings should be drawn in Indian ink. We regret that we must ask authors to pay for making blocks, if this is necessary.

J. W. ARROWSMITH LTD., WINTERSTOKE ROAD,
BRISTOL.

### *Notes and News*

Dr. Joseph Harold Sheldon received the honorary degree of Doctor of Medicine on July 3rd, 1956. Dr. Sheldon's best known works are his monographs "Haemachromatosis" and "Social Medicine of Old Age". This honorary degree was conferred last in 1938 on Sir William George Savage.

Mr. A. L. Eyre-Brook, on the invitation of the Secretary of State for the Colonies, is on a ten week visit to the Western Pacific territories as Consultant Adviser to the Colonial Medical Service.

Dr. T. E. Oppé, M.R.C.P., D.C.H., has been appointed Lecturer in Child Health.

Dr. D. Bowden has been appointed Assistant Professor of Pathology at St. Louis University, Missouri.

Dr. David W. Pugh and Dr. J. E. Cates have been elected Fellows of the Royal College of Physicians.

### UNIVERSITY OF BRISTOL

*Doctor of Medicine, Honoris Causa*, Dr. Joseph Harold Sheldon.

### *M.B., Ch.B.* (*June*, 1956)

WITH SECOND-CLASS HONOURS

Pauline Frances Haswell, (with distinction in Public Health).
Dennis Howard Wright.

PASS

Miriam Mary Alderwick.

Babatunde Oladipo Amure.

Elizabeth Margaret Forsythe Andrews.

John Henry Andrews.

John Christopher Bailey.

Edwin Peter Beck.

William Edward Benny, (with distinction in Public Health).

Brian Thomas Allaway Clarke.

Ruth Ethel Ashurst Coles.

Harry Edward Cramp.

John David Davison.

Keith Morton Evans.

Irene Elizabeth Gibbs.

Paul Michael Gill.

Claude Louis Godfrey Hale.

Arthur Frederick Hickson.

Alex Leonard Horton.

Rachel Mary Hulbert.

Alan Thomas Lloyd.

Sheila Margaret McClements.

June Margaret McLeod.

Terence Morley.

Richard Walford Morris.

Anezionwu Nwankwo Okoro.

Kenneth Michael Parry.

Weekakkodige Fredrick Perera.

Clement Keith Protheroe.

Michael John Rose.

William John Stephen.

Stuart James Strong.

Jeremy Philip Telling.

Michael James Watson.

Anthony Harold Elvin Williams.

Mary Bowen Wilson.

Christopher Holman Wood.

### PRIZES

*Edward Fawcett Memorial Prize for* 1956: Miss S. J. Smith.

*The Foyle Prize for* 1956: Miss M. J. Smith.

*Richard Clarke Prize in Child Health:* Mr. D. H. Wright.

*Russell Cooper Prize:* Mr. D. E. Bowden.

*The Thomas Edgeworth and Francis Henry Edgeworth Prize in Physiology:* Mr. J. R. Winslade.

*Tibbits Memorial Prize for* 1956: Mr. D. H. Wright.

*William Hyde Award* (£300 p.a.): Dr. Martin Herford.

Given by Research Board for the Correlation of Medical Science and Physical Educational Studies of mental and physical problems of young people leaving school and entering industry.

### EXAMINATION RESULTS

*M.R.C.P.* (*London*). J. S. Bearcroft, M. S. Dunnill.

### Appointments

### UNITED BRISTOL HOSPITALS

### MARCH—MAY, 1956

| *Name* | *Appointment* | *Formerly* |
|---|---|---|
| ROBERTS, DOUGLAS IVOR, M.B., B.S., F.R.C.S.(Eng.) | Senior Surgical Registrar | Surgical Registrar, U.C.H., and Clinical Assistant, St. Paul's Hospital. |
| FREEMAN, ANTHONY GEORGE, M.A., M.D., M.R.C.P. | Senior Medical Registrar (exchange of appointment with Dr. Crow) | Senior Medical Registrar, Exeter Clinical Area. |
| YELL, JOAN, M.B., B.S., M.R.C.P. | Medical Registrar | Medical Registrar, Dryburn Hospital, Durham. |
| ALMS, MICHAEL, M.B., F.R.C.S., M.CH.ORTH. | Senior Orthopaedic Registrar | Orthopaedic Surgeon to Colony of Mauritius. |
| ROFFEY, PETER JAMES, M.B., B.CHIR., F.F.A.R.C.S., D.A. | Senior Anaesthetic Registrar | Anaesthetic Registrar, Hammersmith Hospital and Postgraduate Medical School. |

### SOUTH WESTERN REGIONAL HOSPITAL BOARD

### BRISTOL

| *Name* | *Appointment* | *Formerly* |
|---|---|---|
| SLEIGH, BEATRICE E., M.B., CH.B.(MANCHESTER–, D.A.(ENG)., F.F.A.R.C.S. | Senior Anaesthetic Registrar, Frenchay Hospital, Bristol. | Anaesthetic Registrar, Frenchay Hospital, Bristol. |
| CHANDLER, C. C. D., M.B., B.S.(LOND)., D.A., F.F.A.R.C.S. | Anaesthetic Registrar, Frenchay Hospital, Bristol. | Anaesthetic Registrar, United Bristol Hospitals. |
| ROBINSON, RUTH M., M.B., B.S.(LOND.), PRIMARY F.R.C.S.(ENG.). | Surgical Registrar, Weston-super-Mare General Hospital. | Locum tenens S.H.O. in Surgery at Weston-super-Mare General Hospital. |
| GALLA, T., M.B., CH.B. (EDIN.), D.P.M. (R.C.P. & S.) | Assistant Psychiatrist, Bristol Mental Hospitals. | Senior Registrar, Bristol Mental Hospitals. |

### NORTH GLOUCESTERSHIRE

| *Name* | *Appointment* | *Formerly* |
|---|---|---|
| ENGLAND, E. J., M.B., B.S. (ADELAIDE), Primary F.R.C.S.(ENG.). | Surgical Registrar, Gloucestershire Royal Hospital, Gloucester | Locum tenens Surgical Registrar at same hospital. |
| READER, D. M., M.B., B.S. (LOND.), D.(OBST.)R.C.O.G. | Registrar in Obstetrics and Gynaecology, Gloucestershire Royal Hospital, Gloucester. | Gynaecological House Surgeon, Birmingham & Midland Hospital for Women. |

### BATH

| *Name* | *Appointment* | *Formerly* |
|---|---|---|
| BERNSTEIN, CYRIL, M.A. (CAMBRIDGE), M.R.C.S., L.R.C.P. | Medical Registrar, Bath Group of Hospitals. | Locum tenens Medical Registrar St. Martin's Hospital, Bath. |
| WATT, P. A., M.A., M.B., B.CHIR., D.(OBST.) R.C.O.G. | Medical Registrar, Bath Group of Hospitals | Locum tenens Medical Registrar Royal United Hospital, Bath. |
| CONWAY, H. J., M.B., B.CH., B.A.O., D.P.M.(R.C.S.I.) | Assistant Psychiatrist, Roundway Hospital, Devizes. | Registrar, Parkside Hospital, Macclesfield. |

### SOUTH SOMERSET

| Name | Appointment | Formerly |
|---|---|---|
| BARRY, D. M. R., B.A., M.B., B.CH., B.A.O., L.M. (ROTUNDA), D.M.R.(D.). | Consultant Radiologist, South Somerset Clinical Area. | Consultant Radiologist, Bolton Royal Infirmary |

### DEVON AND CORNWALL

| Name | Appointment | Formerly |
|---|---|---|
| BURDON, J. F., M.B., B.S. (DUNELM). | General Practitioner Anaesthetist, Paignton Hospital. | In general practice, Paignton. |
| MANSFIELD, G. C., M.B., CH.B. (GLASGOW), D. (OBST.)R.C.O.G. | General Practitioner Anaesthetist, Paignton Hospital. | In general practice, Totnes. |
| TROBRIDGE, G. F., M.B., CH.B. (B'HAM), M.R.C.P. (EDIN.). | Assistant Chest Physician, Hawkmoor Chest Hospital. | Registrar, Hawkmoor Chest Hospital. |
| CANTRELL, G. L., M.B., CH.B. (MANCHESTER), D.O. (LONDON). | Consultant Ophthalmic Surgeon, Exeter Clinical Area. | Senior Registrar in Ophthalmology, United Bristol Hospitals. |
| CRAFT, M. J., M.B., B.S. (LOND.), M.R.C.P. (EDIN.) | Deputy Medical Superintendent, Royal Western Counties Instn. Starcross. | Registrar, Bethlem Royal & Maudsley Hospitals, London. |
| WEEKS, K. F., M.B., B.S. (LOND.), D.P.M. | Assistant Psychiatrist, Moorhaven Hospital, Ivybridge. | Senior Registrar, Warlingham Park Hospital. |

